# Turbulent current sheet frozen in bursty bulk flow: observation and model

**DOI:** 10.1038/s41598-022-19266-6

**Published:** 2022-09-15

**Authors:** L. Q. Zhang, Chi Wang, L. Dai, W. Baumjohann, James L. Burch, Yu. V. Khotyaintsev, J. Y. Wang

**Affiliations:** 1 State Key Laboratory of Space Weather (National Space Science Center, Chinese Academy of Sciences), Beijing, 100080 China; 2 Space Research Institute, Austrian Academy of Sciences, 8042 Graz, Austria; 3 Southwest Research Institute San Antonio, San Antonio, TX 78238 USA; 4 Swedish Institute of Space Physics, Uppsala, Sweden; 5 Information Engineering College, Central University for Nationalities, Beijing, 100081 China

**Keywords:** Space physics, Magnetospheric physics

## Abstract

Utilizing four-point joint observations by Magnetospheric Multiscale Spacecraft (MMS), we investigate the main features of the current sheet frozen in (CSFI) the bursty bulk flow. Typical event on the steady long-lasting BBF on July 23, 2017 shows the enhanced dawn-dusk current (Jy_0_) in the CSFI (β ~ 10). The magnitude of the Jy_0_ in the CSFI is about 5.5 nA/m^2^. The CSFI is highly turbulent, with the ratio of ∆J/J_0_ of ~ 2 (where ∆J is perturbed J). The turbulent CSFI is characterized by intermittent current coherent structures. The magnitude of the spiky-J at coherent structures is typically above 30 nA/m^2^. Spectrum analysis exhibits that BBF turbulence follows distinct dissipation laws inside and outside the CSFI. Based on MMS observations, we propose a new model of the BBF in the framework of magnetohydrodynamics. In this model, the BBF is depicted as a closed plasma system with the localized current sheet frozen at the center of the flow (Taylor’s hypothesis). In the light of principle of Helmholtz-decomposition, the BBF motion in the tail plasma sheet is explained. The model also predicts the thermal expansion of the BBF after leaving the reconnection source region.

## Introduction

Bursty bulk flow (BBF) is a common phenomenon in the Earth’s magnetotail^1–3^. It is widely accepted that the BBF is generated by near-Earth magnetic reconnection^4,5^. BBF undertakes the main task of energy transport in the tail plasma sheet. As the main energy carrier, plenty of energy would be released during BBF deceleration. Part of flow energy is converted into magnetic energy piled up in the deceleration region^6,7^. Part is converted into wave energy propagating away from deceleration region^8,9^. Many magnetospheric activities are linked to BBF deceleration, such as magnetic dipolarization in the near-Earth tail region^10,11^, Alfvénic auroral formation in the ionosphere^12,13^, and Pi2 on the ground^14^.

Recent observations show growing evidence of the BBF as a complex plasma flow^15–18^. Firstly, the BBF has a complex flow structure. The direction of the flow relative to magnetic field has a gradual transition from predominantly perpendicular in the current sheet to predominantly parallel at the plasma sheet boundary layer (PSBL)^19^. Secondly, the BBF is almost permanently turbulent. Utilizing four-point observation from MMS spacecraft, Zhang et al. investigated in details the vorticity (${{\varvec{\upomega}}} = \nabla \times {\mathbf{V}}$ ) within the BBF^20–22^. They found that the strength of the ω-field depends highly on the BBF velocity. The higher the BBF velocity, the stronger the vorticity. In addition, the ω-field of the BBF has strong anisotropy. The ω-field is predominantly perpendicular, both in the current sheet and near the PSBL.

Historically, the BBF is explained by the depleted flux tube model^23,24^. In the depleted flux tube model, the flux tube is closed with two footpoints located at the ionosphere. The depleted flux tube model is initially developed to solve the problem of “pressure disaster”^25^. The “pressure disaster” arises from the large-scale magnetosphere convection, i.e., the slow earthward moving flux tube with typical velocity of several tens kilometer per second^26^. Later, the depleted flux tube is invoked to interpret the fast flow with enhanced normal field in the plasma sheet^27,28^. However, “flux tube” fails to explain the flow structure internal of the BBF, neither earthward flow with positive Bz nor tailward flow with negative Bz.

In the present paper, we propose a new physical model of the BBF based on four-point joint observation by Magnetospheric Multiscale Spacecraft (MMS)^29^. While operating in the Earth’s magnetotail, the measurement data has the time-resolution of 4.5-s for Fast Plasma Investigation (FPI)^30^, 0.125-s for fluxgate magnetometers (FGM)^31^, 0.03-s resolution for 3D Electric Field Double Probe (EDP)^32^. Geocentric solar magnetospheric (GSM) coordinates are adopted. Curlometer analysis^33^ from four-point MMS measurements of the magnetic field is used to calculate the current density. Besides, the median filter (cut-off frequency of 0.003 Hz) is used to separate the unperturbed field (${\varvec{B}}_{0}$**/**${\varvec{E}}_{0}$) and the perturbed field (**∆B/∆E**). Then, the perturbed fields are used to calculate Poynting vector (${\mathbf{P}} = {{\varvec{\Delta}}}{\mathbf{E}} \times {{\varvec{\Delta}}}{\varvec{B}}$). In calculations of the Poynting flux, both electric and magnetic fields are interpolated to match the 4.5 s time cadence of the FPI data. Typical event confirms the enhanced dawn-dusk current in the current sheet frozen-in (CSFI) the BBF. Our BBF model highlights the potential significance of the CSFI on the nonlinear energy cascade of the BBF turbulence. On the basis of Helmholtz-composition principle, the motion of the BBF in the tail plasma sheet and its acceleration/deceleration is explained. Besides, the model predicts the thermal expansion of the BBF after its leaving reconnection source region.

### Theoretical fundamental

The Helmholtz decomposition is a fundamental theorem in fluid analysis^34,35^. In the light of Helmholtz-decomposition principle, the velocity of any fluid element can be decomposed into: ${\mathbf{V}} = {\varvec{V}}_{0} + {{\varvec{\upomega}}} \times {\mathbf{\delta r}} + {{\varvec{\upvarepsilon}}} \cdot {\mathbf{\delta r}}$. Thus, the velocity field is separated into an irrotational (V_0_) and rotation (ω) parts. In the plasma environment, the velocity of fluid element: ${\text{V}} = \frac{{{\varvec{m}}_{{\varvec{i}}} {\varvec{V}}_{{\varvec{i}}} + {\varvec{m}}_{{\varvec{e}}} {\varvec{V}}_{{\varvec{e}}} }}{{{\varvec{m}}_{{\varvec{i}}} + {\varvec{m}}_{{\varvec{e}}} }} \approx {\varvec{V}}_{{\varvec{i}}}$, where ${\text{V}}_{{\text{i}}}$ and ${\text{V}}_{{\text{e}}}$ are the convective velocity of ion and electron, and $m_{{\text{i}}}$ and $m_{e}$ are the mass of ion and electron. Applying Helmholtz-decomposition principle to plasma flow, the velocity of a fluid element is that:

1 $$ {\varvec{V}}_{{\varvec{i}}} = {\varvec{V}}_{{{\varvec{i}}0}} + {{\varvec{\upomega}}}_{{\varvec{i}}} \times {\mathbf{\delta r}} + {{\varvec{\upvarepsilon}}}_{{\varvec{i}}} \cdot {\mathbf{\delta r}} $$

where ${\varvec{V}}_{{{\varvec{i}}0}}$ is the translation velocity of ion flow, (${{\varvec{\upomega}}}_{{\varvec{i}}} = \nabla {\varvec{V}}_{{\varvec{i}}}$) is ion vorticity, and ${{\varvec{\upvarepsilon}}}_{{\varvec{i}}}$ is the ion transformation tensor coefficient. At MHD scale, there has ${\mathbf{V}}_{{\varvec{i}}} = {\mathbf{V}}_{{\varvec{e}}}$ and ${{\varvec{\upomega}}}_{{\varvec{i}}} = {{\varvec{\upomega}}}_{{\varvec{e}}}$. Here,${{\varvec{\upomega}}}_{{\varvec{e}}}$

According to magnetohydrodynamic (MHD) theory, the translation velocity ${\varvec{V}}_{{{\varvec{i}}0}}$ in Eq. (1) obeys the motion equation

2 $$ \begin{aligned} {\uprho }\frac{{{\text{d}}{\varvec{V}}_{{{\varvec{i}}0}} }}{{{\text{dt}}}} & = {\mathbf{J}} \times {\mathbf{B}} - \nabla {\text{P}} \\ & = - \nabla \left( {{\text{P}} + \frac{{B^{2} }}{{2{\upmu }_{0} }}} \right) + \frac{{\left( {{\mathbf{B}} \cdot \nabla } \right){\mathbf{B}}}}{{2{\upmu }_{0} }} \\ \end{aligned} $$

The first and second terms at the right hand are the gradient of the plasma pressure and magnetic tension force, respectively. This equation decides the flow acceleration/deceleration process.

### Case study

**Figure 1:**
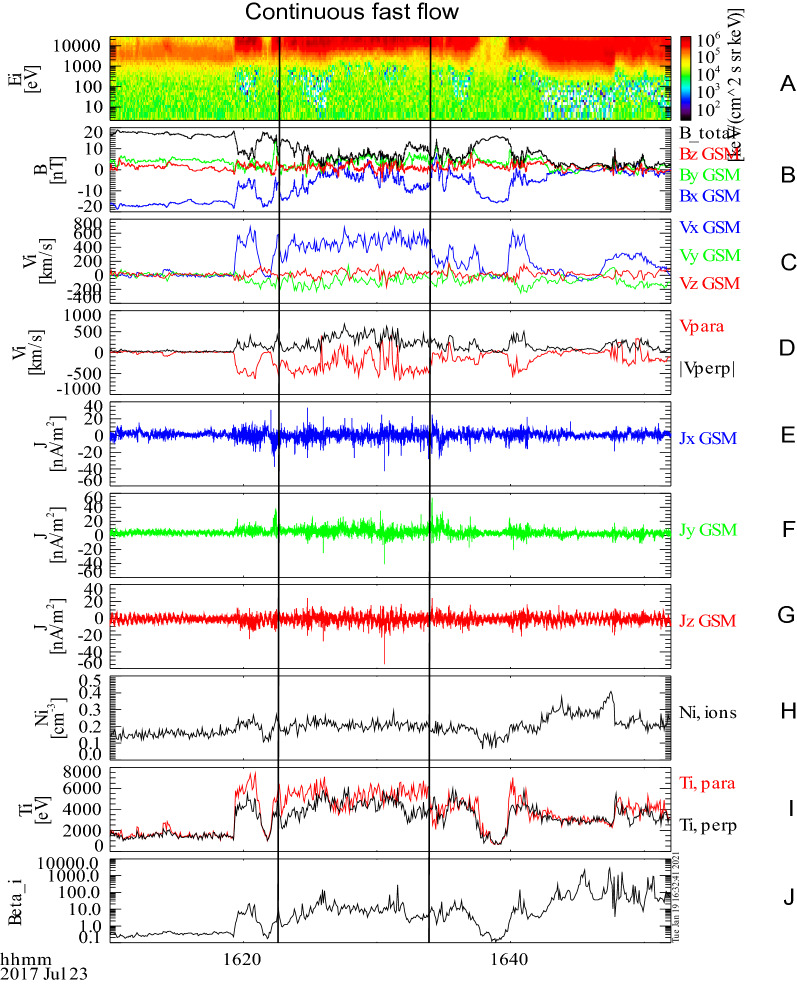
Continuous fast flow embedded turbulent current sheet on 23 July, 2017 by MMS1 spacecraft (GSM coordinates). (**A**) plots ion energy spectrum. (**B**) shows measured Bx, By, Bz, and B total. (**C**) is measured Vx, Vy, and Vz. (**D**) is parallel velocity (V_//_) and perpendicular velocity (V_⊥_). (**E**–**J**) are the three components of the current density (calculated by **J** = ∇ × **B**∕*μ*_0_). (**H**,**J**) show ion density (n) and temperature (T). Panel J is plasma β (ratio of thermal pressure to magnetic pressure).

A steady long-lasting BBF is recorded on July 23, 2017 by MMS1 spacecraft. Associated evolutions of the plasma and field from 16:10 to 16:50 UT are shown in Fig. 1. Prior to the flow, MMS1 is posited at the boundary layer of the plasma sheet (β ~ 0.2). The BBF appears at 16:19 UT. After entering into the BBF, MMS1 rapidly moves into the current sheet. Ion temperature increases from ~ 1.7 to ~ 4.5 keV and ion density increases from 0.15 to 0.2 cm^−3^. Correspondingly, high-energy ion flux exhibits a prominent enchantment above 10 keV (Panel A).

From 16:23 to 16:35 UT (marked by the two vertical lines), the flow is quite steady. The flow velocity is ~ 500 km/s which is above Alfvénic velocity (V_A_ = 455 km/s). The super-V_A_ BBF, with small Vy and Vz components, is slightly fluctuated. During this interval, MMS keeps staying in the current sheet (β ~ 10). At 16:38 UT, MMS1 shortly dips into the boundary layer of the plasma sheet. Then, it turns back to the flow. After the flow pass-by, the current sheet recovers to be quiet. Comparing to the CSFI, the post-BBF BCS has a higher density but a lower temperature.

**Figure 2:**
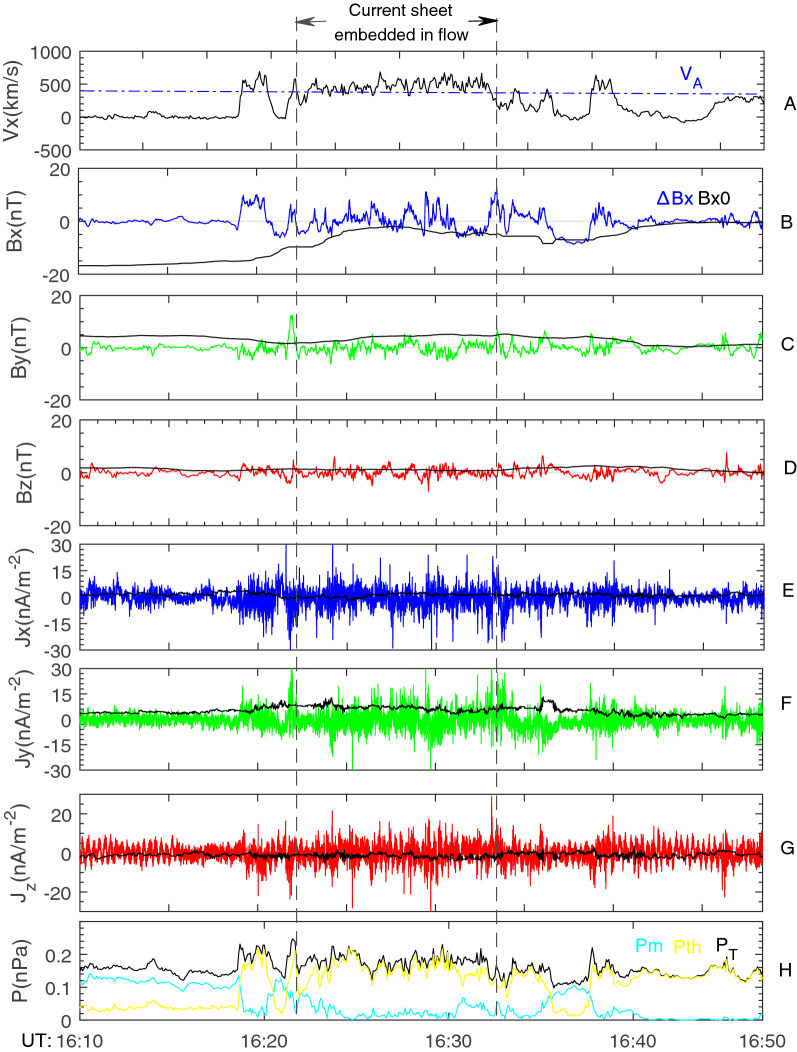
Turbulent current sheet without Bz-enhancement embedded in the earthward-traveling fast flow (same interval as Fig. 1). (**A**)V_x_. (**B**) Perturbed Bx (∆B_x_) and unperturbed Bx (B_x0_) (from a 5-min low-pass filter). (**C**) ∆B_y_ and B_y0_. (**D**) ∆B_z_ and B_z0_. (**E**) J_x0_ (calculated by unperturbed field). (**F**) J_y0_. (**G**) J_z0_. (**H**) Thermal pressure (P_th_), magnetic pressure (P_m_), and total pressure (P_T_).

Current variations in the CSFI and BCS are plotted in Fig. 2. The Bz0 in the CSFI is quite small and the normal field of the CSFI is illegible. The CSFI is characterized by a positive J_y0_ (within two vertical lines). This confirms the dawn-dusk current in the CSFI. The magnitude of the J_y0_ in the CSFI is about 5.5 nA/m^2^, which is much higher than the BCS. The CSFI is highly turbulent. The amplitude of the perturbed current (∆J) is ~ 10 nA/m^2^, and the ratio of ∆J/J_0_ is close to 2. The turbulent CSFI is characterized by intermittent coherent structures^36^. Typically, the magnitude of the spiky current at coherent structures is above 30 nA/m^2^.

Panel 2(H) shows pressure variation inside and outside the flow. The BBF is characterized by higher thermal pressure (P_th_) and lower magnetic pressure (P_m_). The total pressure is higher inside the flow than outside the flow. This implies the ongoing thermal expansion of the BBF. The P_E_ in the pre-flow plasma sheet and P_T_ in the post-flow plasma sheet are almost equal. As a consequence, the pressure at the two sides of the flow is basically balanced.

**Figure 3:**
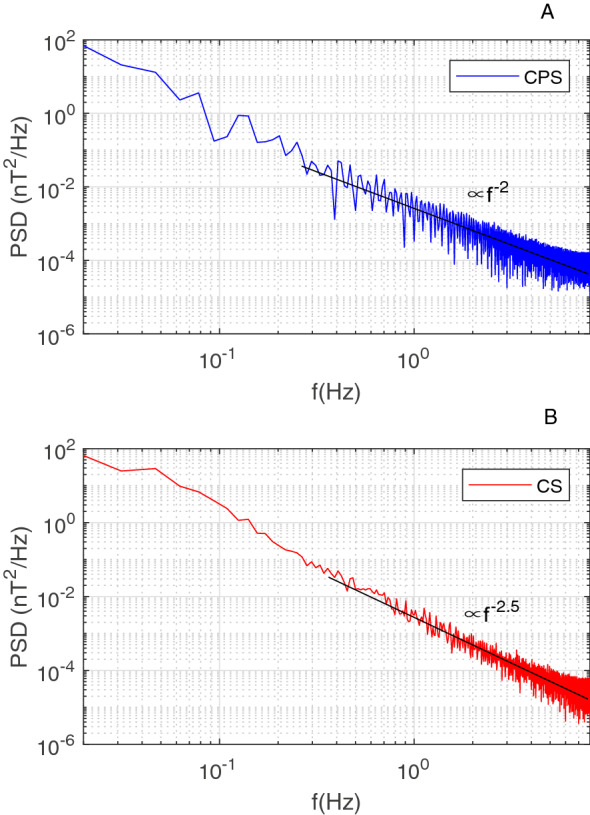
Power spectrum distribution of the turbulent B during the interval of the reconnection jet from 16:23 to 16:35 UT.

Figure 3 exhibits the B-spectrum inside CSFI (B < 10 nT) and outside CSFI (B > 10 nT). It can be seen that the BBF turbulence follows different law inside and outside the CSFI. Below 0.4 Hz, the two spectra have similar evolutions follow the slope of − 5/3. Above 0.4 Hz, the two spectra spilt into two different slopes. The B-spectrum tends to have a steeper slope in the PS (− 2-like) than in the CSFI (− 2.5-like).This implies a faster energy transfer and dissipation toward small scale inside the CSFI than outside the CSFI.

Physically, the CSFI can be treated as the current vortex sheet^37,38^. The current vortex sheet supports both fluid-type (Kelvin–Helmholtz) instability and non-fluid (tear-mode) instability. In this sense, the CSFI behaves as the boundary layer internal of the flow and contributes to mediate the nonlinear energy cascade process of the BBF turbulence. The distinct power laws inside and outside the CSFI could be related to the different BBF cascade near and far away from the current boundary layer.

### Statistical result

Case study shows the potential thermal expansion of the BBF. The thermal evolution of the BBF depends only on the difference of the plasma pressure inside and outside the flow, The BBF expands if Pin > Pout, and contracts if Pin < Pout. The thermal expansion has substantial effect on the evolution of the BBF. Firstly, the thermal expansion decides the spatial scales of the BBF. Secondly, the thermal expansion dominates the properties of the BBF, including its ion density and temperature. Thirdly, the thermal expansion changes the property of the BBF turbulence from incompressible to compressible.

**Figure 4:**
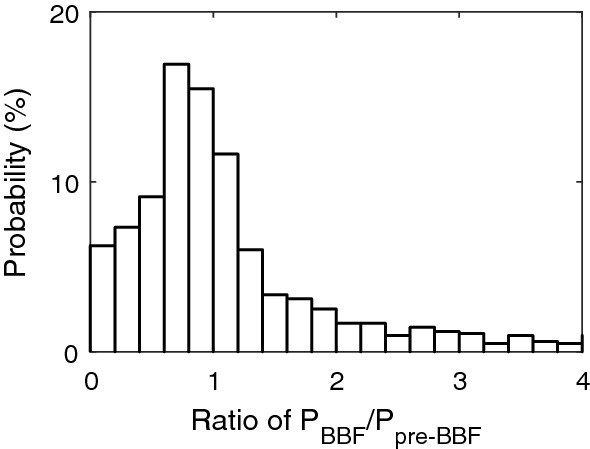
Ratio of P_BBF_ to P_BPS_.

Utilizing MMS data collected from May 2017 to Oct 2018, we perform a statistical and comparative study on the plasma pressure of the BBF with that of the BPS. The BBF is selected by the criterion of the duration of V_⊥x_ > 200 km/s for longer than 20 s. The selection region is confined in the box of -25 R_E_ < X < − 10 R_E_, − 15 R_E_ < Y < 15 R_E_ and − 5 R_E_ < Z < 5 R_E_. There are totally 831 BBF events selected. For each BBF, the plasma pressure is averaged over BBF time. The obtained average value is used to be P_in_ of this BBF. For each flow, the plasma pressure in the pre-BBF BPS is averaged over 10 min (before the approach of the BBF). Obtained average value is used to be P_out_ of that BBF. Figure 4 plots BBF P_in_ versus pre-BBF P_out_. The ratio of BBF P to pre-BBF P varies mainly in the range of 0.1 to 5, with the peak at 0.8. In particular, about 15% of the BBFs have a higher ratio than 1.5. This strongly suggests the thermal expanded of the BBF.

## Discussion

With four-point joint observation by MMS spacecraft, we present the different turbulent characteristics of the BBF in the frame of time series and frequency domain. This is very important to understand the mechanics related to our earth environment system as well as space plasma environment. The BBF turbulence is intrinsically the superposition of flow and wave. The interaction between the eddy and wave is unavoidable within the BBF turbulence. Associated studies in the fluid turbulence^39–41^ show that the interaction between eddy and wave could substantially affect the nonlinear energy cascade process. Study on the BBF turbulence is expected to bring new knowledge on the interaction between eddy and wave in turbulence theory.

**Figure 5:**
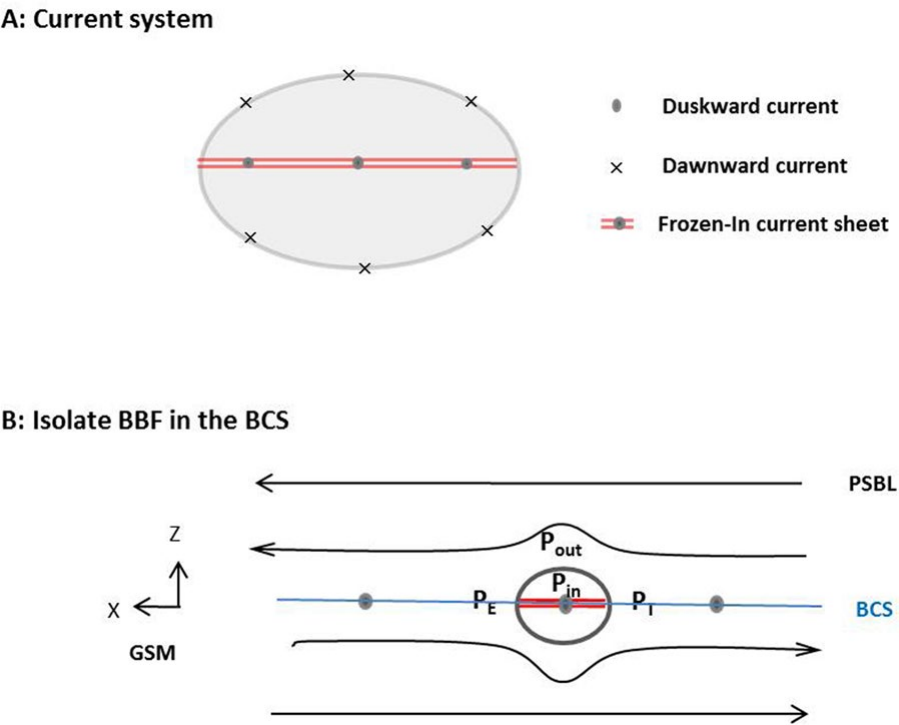
A schematic of current system of the BBF in the CSFI model and its motion in the background plasma sheet. (**A**) View on the closed current system of the fast flow confined current sheet in the meridian profile. (**B**) Motion of the BBF dependence on pressure gradient in the tail plasma sheet.

To further study the BBF turbulence, the primary thing is to construct the proper fluid model of the BBF. The key is to reconcile the main body of the flow with the current sheet frozen in the flow^42,43^. In classical MHD theory, the electric current is always closed ($\nabla \cdot {\mathbf{J}} = 0$). Thus, the enhanced dawn-dusk current in the localized current sheet frozen in the BBF must be closed. A natural way to close it is via electric current at the surface of the flow. Figure 5A shows the closed current system of a BBF in the meridian profile. At the dusk-side boundary of the BBF, the cross-tail current is split into two equivalent branches. One branch flows along the top of the BBF. The other flows along the bottom of the BBF. The two branches reach to the dawn-side boundary where they meet together and turn back to the current sheet. The closed current system separates the BBF from the background plasma and magnetic field. In this way, the BBF forms an isolate plasma system traveling in the background plasma sheet.

Now, we consider the BBF motion in the plasma sheet. The motion of the BBF in the background plasma sheet is illustrated in Fig. 5B. We know that the motion of the bulk flow could be simplified to the motion of the mass center. Assuming that the CSFI has small normal component (the Harris-type current sheet as observed in this study)^43,44^, the magnetic tension force (quantified by ${ }{\mathbf{J}}_{Y} \times {\mathbf{B}}_{z}$) in Eq. 3) could be neglected. Thus, the motion of the BBF, its acceleration and/or deceleration, depends only on the plasma pressure gradient at the two sides of the flow. If the background current sheet (BCS) has a positive pressure gradient (P_E_ > P_T_), the BBF would be decelerated. Vice versus, if there has P_E_ < P_T_, the BBF would be accelerated.

As a summary, we propose a new physics model of the BBF, i.e., the CSFI model. In this model, the BBF is depicted as a closed plasma system with the localized current sheet frozen at the center of the flow. The model highlights the contribution of the CSFI to mediate the nonlinear energy cascade process of the BBF turbulence. Finally, it is worthy to point out that the CSFI-BBF model is applicable to the popular reconnection jet in the Sun-Earth space, such as the surface of the Sun and the magnetosheath region downstream the bow shock as well.
